# The paradigm of intracellular parasite survival and drug resistance in leishmanial parasite through genome plasticity and epigenetics: Perception and future perspective

**DOI:** 10.3389/fcimb.2023.1001973

**Published:** 2023-02-06

**Authors:** Mohd. Kamran, Rahul Bhattacharjee, Sonali Das, Sohitri Mukherjee, Nahid Ali

**Affiliations:** Infectious Diseases and Immunology Division, Indian Institute of Chemical Biology, Kolkata, West Bengal, India

**Keywords:** leishmania, plasticity, parasite survival, drug resistance, genome organization

## Abstract

*Leishmania* is an intracellular, zoonotic, kinetoplastid eukaryote with more than 1.2 million cases all over the world. The leishmanial chromosomes are divided into polymorphic chromosomal ends, conserved central domains, and antigen-encoding genes found in telomere-proximal regions. The genome flexibility of chromosomal ends of the leishmanial parasite is known to cause drug resistance and intracellular survival through the evasion of host defense mechanisms. Therefore, in this review, we discuss the plasticity of *Leishmania* genome organization which is the primary cause of drug resistance and parasite survival. Moreover, we have not only elucidated the causes of such genome plasticity which includes aneuploidy, epigenetic factors, copy number variation (CNV), and post-translation modification (PTM) but also highlighted their impact on drug resistance and parasite survival.

## 1 Introduction

Leishmaniasis primarily a zoonotic poverty-driven neglected tropical disease largely overlooked by the pharmaceutical industry (Bray, 1974), is usually transmitted to the human host by the bite of a sandfly. *Leishmania* amastigotes enter the gut of the sandfly after the bloodmeal wherein they develop into pro-cyclic promastigotes. These promastigotes divide and replicate to form the metacyclic promastigotes after 4-7 days. During this stage, they migrate to the pharynx of the sandfly and after its bite, the promastigotes get inside the host body to get phagocytized by the macrophage. Once inside the phagosome, they differentiated into amastigotes. When the sandfly withdraws blood from the mammalian host the parasite cells get inside its gut where it again gets transformed into the promastigote form and thus the cycle continues (Bates, 2018).

Even though leishmaniasis is present all over the world, the disease burden in humans is concentrated in certain parts only. Visceral leishmaniasis (VL) is mainly relevant in India, Brazil, Sudan, Somalia and Kenya. Despite its endemicity in these countries, new cases tend to arise and approximately 500,000 have been reported in rural parts of South Asia, Brazil, and parts of Africa. VL, like all leishmaniases, affects mostly the rural populations. For decades VL has caused epidemics in Bihar, one of the poorest and most densely populated states in India (Bi et al., 2018). Cutaneous leishmaniasis (CL) is mainly relevant in Iran, Syria, Pakistan, Saudi Arabia, Algeria and Peru (Roatt et al., 2014). Kabul is considered to be the epicenter of CL in the world with 67,000 new cases per year (Burza et al., 2018). Currently, there is no approved vaccine against this disease, and the majority of control strategies involve therapeutics (Handman, 2001; Murray et al., 2005; Palatnik-De-Sousa, 2008). In India, antimonial based medications for treating the disease is disused due to the development of resistance against these formulations. However, pentavalent antimony (Sb) the standard treatment for the last 70 years remains the backbone in many regions of *Leishmania* endemicity. The polyene antibiotic amphotericin B (AMB), which has been proven to be 95% effective against VL in India is another first-line therapy (Sundar et al., 2010).

Even though Liposomal AMB requires intravenous delivery, still it has become a common treatment in many countries. However, there were geographical disparities in the response of liposomal AMB, with VL cases in India responding better than those in East Africa or South America (Berman et al., 1998; Saravolatz et al., 2006). Clinical AMB resistance is uncommon, and parasites were found to be sensitive even after numerous treatments in the same patient (Lachaud et al., 2009). Miltefosine (MTF) is an alkyl-lysophospholipid analog administered orally to treat leishmanial parasites (Croft et al., 1987; Jha et al., 1999). Since its introduction in India in 2002, miltefosine has been successfully used to treat VL but the relapse rates have increased owing to MTF resistance (Sundar et al., 2002; Zuckerman et al., 2012; Burza et al., 2013). Aminoglycoside paromomycin (PMM) has been licensed for the treatment of VL in India (Jha et al., 1998; Sundar et al., 2007). So far, the restricted use of PMM has prevented the establishment of resistance. However, there have been geographical differences in PMM efficacy against VL between East Africa and India (Hailu et al., 2010; Musa et al., 2010). Finally, due to severe toxicity, pentamidine (PTD) has been discontinued for the treatment of VL and is now only used in the treatment of CL in South America (Soto et al., 1994; Lai a Fat et al., 2002; Roussel et al., 2006; Bhattacharyya et al., 2022). The mode of action of antimonials is unknown despite its use for more than six decades. It has been reported to cause the generation of reactive oxygen species, trypanothione depletion, and apoptosis-like death, although an exact mechanism is yet to be determined (Sereno et al., 2001; Lee et al., 2002; Sudhandiran and Shaha, 2003; Wyllie et al., 2004; Mehta and Shaha, 2006; Mookerjee Basu et al., 2006; Mandal et al., 2007; Vergnes et al., 2007; Moreira et al., 2011). Thus, there is a considerable need for new anti-leishmanial drugs. Further understanding of genome organization would aid in designing of targeted therapies against drug resistance in *Leishmania*.

The *Leishmania* sp. uses various strategies to adapt to changing environment while shifting between different hosts. These include a shift in the collection of expressed genes, mosaic aneuploidy, and copy number variation. The copy number variation (CNV) causes variation in host-parasite interaction for cellular invasion and immune evasion mechanisms causing genomic and phenotypic heterogeneity (Reis-Cunha et al., 2018). Furthermore, CNV produces extrachromosomal circular or linear amplified DNAs by amplification of chromosomes, affecting entire chromosomes or specific genomic areas for mosaic aneuploidy. Mosaic aneuploidy may be viewed as a potent intracellular parasite survival strategy causing enhanced genomic variability and a population clearance of heterozygous cells without altering genetic heterogeneity (Sterkers et al., 2012). Moreover, the changes in environmental variables cause phenotypic variability which leads to drug resistance. Thus, the capacity of leishmanial parasites to respond to the pharmacological inhibition *via* CNVs and mosaic aneuploidy led to the development of genomic screens like Cos-Seq, and FISH to speed up drug targets for novel prospective therapeutic candidates (Grünebast and Clos, 2020).

**Figure 1:**
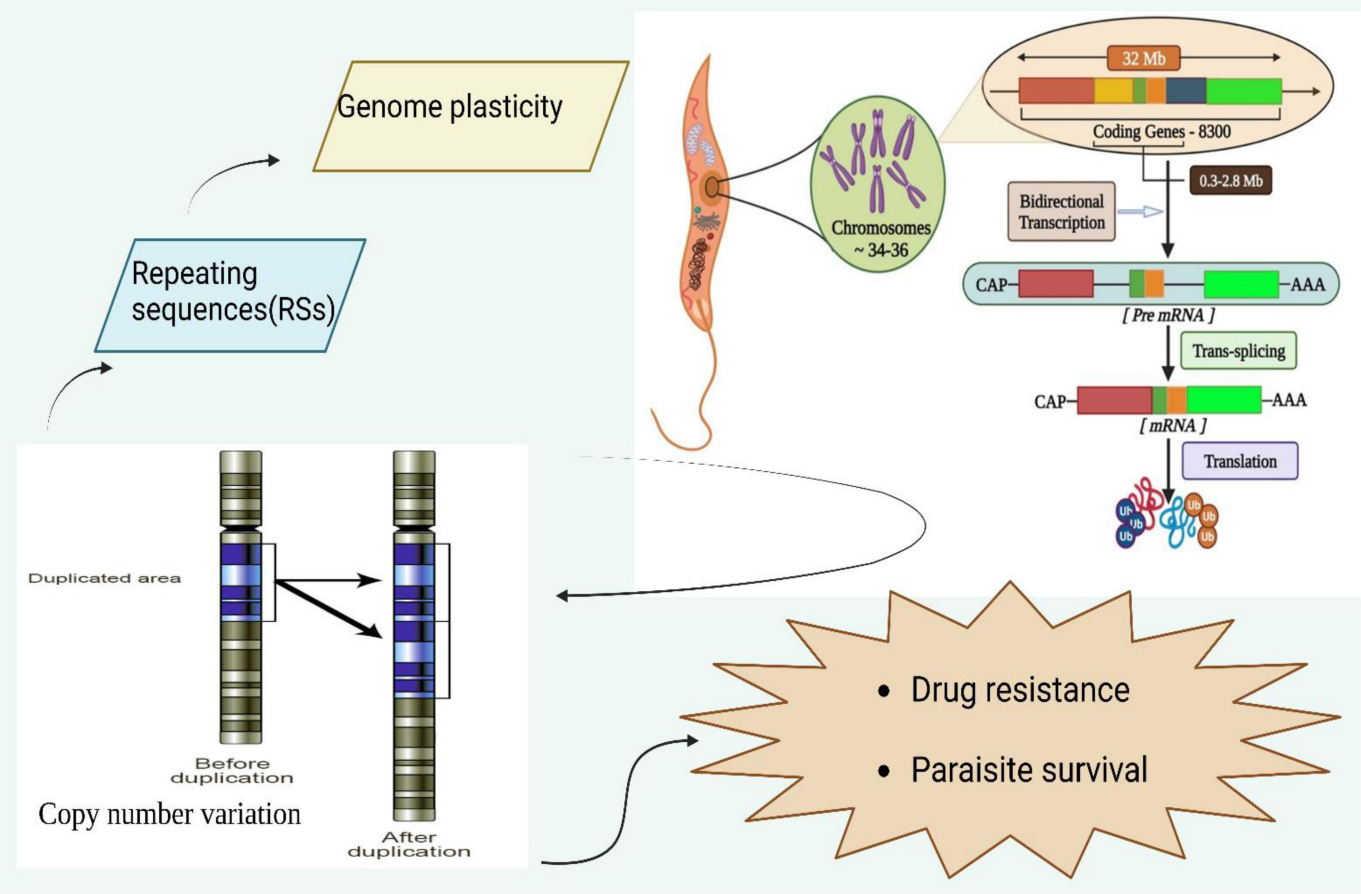
Genomic organization of leishmanial parasites and its regulation of Copy number variation to modulate and influence drug resistance and parasite survival. The leishmanial species contains 34-36 chromosomes where a vast polycistronic unit spans through the protein-coding genes with a size from 0.3 to 2.8 mb. The transcription of these lengthy polycistronic units occurs bi-directionally from the start sites situated at switch regions of the strand.

Thus, in this review, we have discussed the regulation of genome plasticity *via* CNV and its impact on drug resistance and intracellular parasite survival (**Figure 1**). Moreover, we not only highlighted the influence of PTMs on genome plasticity for parasite survival through environmental factors but also elucidated the role of epigenetics in parasite growth.

## 2 Genome organization

The *Leishmania sp* genome is approximately 32 mb in size containing around 8,300 coding genes (Ivens et al., 2005; Peacock et al., 2007). More than 99% of genes in the *Leishmania* genus are conserved with only a few species-specific genes found altogether in *L. major*, *L. infantum*, and *L. braziliensis* (Peacock et al., 2007). The leishmanial species contains 34-36 chromosomes where a vast polycistronic unit spans through the protein-coding genes with a size from 0.3 to 2.8 mb (Wincker et al., 1996; Britto et al., 1998; Martínez-Calvillo et al., 2003; El-Sayed et al., 2005; Ivens et al., 2005; Peacock et al., 2007; Raymond et al., 2012). The transcription of these lengthy polycistronic units occurs bi-directionally from the start sites situated at switch regions of the strand in the absence of RNA polymerase-II promoters (Martínez-Calvillo et al., 2004). The attachment occurs through the trans-splicing of nucleotides at the long leader RNA of the 5’end of each mRNA with simultaneous polyadenylation at the 3’end for processing of mRNA (Matthews et al., 1994; Haile and Papadopoulou, 2007). The mRNA stability and translational rate of the leishmanial parasite depends on 3′-untranslated regions (3′-UTRs) to regulate gene expression *via* altered environmental factors for its development due to its lack of transcriptional control (Bringaud et al., 2007; Dupé et al., 2014). *Leishmania* frequently uses DNA copy number variations (CNVs) (aneuploidy, gene amplification, or gene deletion) to regulate the expression of drug targets, drug transporters, or other determinants of resistance to overcome drug pressure (Gordon et al., 2012; Pfau and Amon, 2012; Shor and Perlin, 2015; Rutledge and Cimini, 2016). Single-nucleotide polymorphisms (SNPs) are caused due to CNVs in pharmacological targets or transporters leading to drug resistance without affecting gene expression (Alwi, 2005).

### 2.1 Regulation of genome plasticity

Leishmanial parasites were previously thought to be diploid but recent research has revealed their aneuploidy (Ubeda et al., 2008a; Leprohon et al., 2009; Downing et al., 2011; Rogers et al., 2011; Sterkers et al., 2011; Mannaert et al., 2012; Sterkers et al., 2012). Aneuploidy patterns were seen among populations of leishmanial parasites at various individual cells. Using mosaic aneuploidy, it was discovered that an average diploid population of a few parasites shared the same ploidy for individual chromosomes when the cumulative ploidy was obtained from next-generation DNA sequencing data (Ubeda et al., 2008a; Sterkers et al., 2011; Lachaud et al., 2014). Variations in chromosomal size were identified across different strains of the related leishmanial parasites (Minning et al., 2011; Reis-Cunha et al., 2015). However, links between increased or decreased chromosome number and drug resistance are circumstantial. This demonstrates that a particular set of genes on the variant chromosomes coordinate to develop resistance. But aneuploidy is a survival mechanism for leishmanial parasites through genomic stability and enhanced virulence in recent years (Sterkers et al., 2012; Sterkers et al., 2014).

The CNV serves as a mechanism for controlling the expression of conditional factors in the absence of transcription initiation and thus establishing a strong link between chromosome ploidy and nucleic acid expression levels (Ubeda et al., 2008a; Leprohon et al., 2009). The mis-aggregation of allele would lead to enhanced chromosomal number leading to overexpression of genes *via* increased copy numbers leading to aneuploidy in the leishmanial parasite. However, the *Leishmania* genome (32 mb) is scattered across 34 to 36 chromosomes and decreased co-expression of genes would cause the parasite to amplify or delete certain smaller portions of DNA as extrachromosomal elements *via* recombination rearrangements at the level of homologous repeating sequences (RSs) (Ubeda et al., 2014).

In a recent study, the full set of RSs in several *Leishmania* species was highlighted, and the entire leishmanial genome was found to possess the ability to alter RSs at the RNA level, causing parasite survival *via* aneuploidy through the generation of extrachromosomal elements (Ubeda et al., 2014). Moreover, LmSIDER1 and LmSIDER2 are two families of SIDER found in *L. major* causing plasticity *via* CNV for parasite survival. SIDER2 destabilizes mRNA and causes mRNA decay, whereas the SIDER1 family regulates mRNA translation in a stage-specific manner (Boucher et al., 2002; Bringaud et al., 2007; Azizi et al., 2017).

## 3 Intracellular host-parasite survival *via* CNV

The genome of *L. infantum* constitutes 2000 RSs for the synthesis of over 3,000 extrachromosomal DNA elements (Ubeda et al., 2014). Short interspersed degenerate retrotransposons (SIDERs), small degenerated retrotransposons, having no specificity for site-specific integration, reside mostly in the vicinity of intergenic regions of directional gene cluster and majority of these elements are located within 3′ UTRs. SIDER elements possess two functions, they either regulate gene expression or act as a structural backbone to facilitate gene rearrangements for CNV of DNA regions (Ouellette and Papadopoulou, 2009; Smith et al., 2009). When leishmanial parasites are exposed to drugs, the extrachromosomal DNA amplifications occur causing amplicons *via* episomes (Ubeda et al., 2008a; Leprohon et al., 2009; Downing et al., 2011; Rogers et al., 2011; Sterkers et al., 2011; Mannaert et al., 2012; Sterkers et al., 2012). The episomes are produced by rearrangements of direct or inverted homologous RSs and are amplified as either circular or linear extrachromosomal DNA (Ubeda et al., 2014). The amplicon production varies with increased drug resistance that allows the parasites to adjust to changes in their environment for parasite survival within the host (Ubeda et al., 2014). Since gene rearrangements are caused by RSs in response to drug resistance, the revelation of newer recombinase proteins involved in rearrangements would lead to preventing drug resistance.

Several drugs cause indirect or direct DNA damage as DSB. The RAD51 is a DNA repair protein that plays a crucial role in homologous recombination (HR) in the intracellular parasite. The double-strand breaks (DSBs) were found to promote RAD51 expression leading to drug resistance in the leishmanial parasite (Mckean et al., 2001; Genois et al., 2012). The inactivation of RAD51 would prevent parasites to synthesize circular extrachromosomal elements but linear amplicons production remained unchanged leading to drug resistance and parasite survival (Ubeda et al., 2014). There are three DNA-repair proteins, that are present in the leishmanial parasite as RAD51 paralogs (RAD51-3, RAD51-4, and RAD51-6) that showed to cooperate for the promotion of homologous recombination (HR) by stimulating RAD51 activity (Genois et al., 2015). The inactivation of RAD51-4 leads to the suppression of the generation of circular amplicons by RSs in an inverted orientation but not linear amplicons in *L. infantum*, when exposed to drugs (Laffitte et al., 2014).

Similarly, MRE11 is a DNA repair nuclease that forms MRN (meiotic recombination) complex with RAD50 as well as NBS1 and is critical for DSB repair by homologous recombination (HR) or non-homologous end joining (Mimitou and Symington, 2011; Genois et al., 2014; Shibata et al., 2014). *L. infantum* constitutes linear amplicons upon treated with drug *via* inverted RSs-causing inactivation of MRE11 for intracellular parasite survival (Laffitte et al., 2014). Furthermore, parasites expressing DNA-binding-proficient but nucleotide-deficient MRE11 only produce circular amplicons during drug selection, indicating that a fully functional MRE11 is required for linear amplification. The inactivation of MRE11 alone or in combination with RAD50 causes significant chromosomal translocation in *L. infantum*, indicating that the MRE11/RAD50 complex is critical for genomic integrity and gene rearrangements (Laffitte et al., 2016a).

Based on the aforementioned studies it could be inferred that modulation of RSs causes the DNA repair protein to promote intracellular host-parasite survival *via* DSBs. Thus, drugs or inhibitory molecules targeting DNA repair proteins could serve as therapeutic interventions to cause regression of leishmanial infection. However, further understanding of other DNA repair proteins modulated by RSs of CNV needs to be done before reaching a definitive conclusion on this aspect. Even though there is a plethora of studies discussing drug resistance and parasite survival, studies focusing on a definite pattern based on genome plasticity are limited and need to be focused on to get a detailed mechanistic overview of intracellular host-parasite survival.

## 4 Intracellular host-parasite survival *via* PTM and epigenetics

### 4.1 Gene expression and translational efficiency

The post-transcriptional regulation of gene expression in the leishmanial parasite is mediated through external environmental stimuli (Brandau et al., 1995; Quijada et al., 1997; Clayton and Shapira, 2007; Grünebast and Clos, 2020). Leishmanial genes are constitutively expressed with no gene-specific transcription control and verified by nuclear run-on and microarray investigations. When axenic amastigotes were compared to promastigotes, a downregulation of RNA production for parasite survival was observed (Leifso et al., 2007; Alcolea et al., 2010). The process of stage differentiation causes enhanced amastigote-specific protein synthesis inside the host body for leishmanial survival (Rosenzweig et al., 2008b; Lahav et al., 2011).

A system-wide RNA abundance study indicated that *Leishmania* gene expression is regulated at levels other than RNA synthesis, such as RNA processing and stability (Clayton and Shapira, 2007). However, changes in mRNA abundance do not always correspond to changes in protein synthesis rates or abundance, indicating the significance of translation efficiency and protein half-life as regulatory targets (Charest et al., 1996; Yao et al., 2002; Decuypere et al., 2012; Azevedo et al., 2015; Bifeld et al., 2018). The RNA half-life poses resistance to antimony as a post-transcriptional mechanism. It can be correlated with the stability of mRNA coding for AQP1 in a 3’-UTR-dependent manner with non-long terminal repeat retrotransposons causing parasite survival and drug resistance *via* plasticity in the leishmanial parasite (Mandal et al., 2015).

### 4.2 Post-translational modifications (PTMs)

Various PTMs including phosphorylation, acetylation, methylation, and glycosylation influence axenic differentiation into amastigotes through modified proteins (Morales et al., 2008; Rosenzweig et al., 2008a; Morales et al., 2010). MAPKs are serine/threonine-specific protein kinases found in all eukaryotes for signal transduction and regulation of gene expression profiles. MAPKs regulate important cellular activities like cell viability, life cycle, morphology, and drug resistance in leishmanial parasites by phosphorylating their substrates (Wiese, 1998; Wiese et al., 2003; Bengs et al., 2005; Kuhn and Wiese, 2005; Wang et al., 2005; Mccarter, 2006; Von Freyend et al., 2010; Cayla et al., 2014). The stability of heat shock proteins and their roles during the life cycle is affected by phosphorylation of HSP70 and HSP90 in MAP kinase 1 of *L. donovani* (Kaur et al., 2017). Moreover, when stationary phase cells were compared with rapidly expanding cells, acetylation levels were enhanced in rapidly growing cells. The acetylation of the H3 variant in the switch region of the telomeric divergent strand causes parasite survival *via* chromatin modification in *L. major* promastigotes (Thomas et al., 2009).

Furthermore, the leishmanial telomeric repeat sequence (GGGTTA) contains glycosylated thymine, known as “Base J” or beta-D-glucosyl hydroxy methyl uracil (Van Luenen et al., 2012; Olinski et al., 2016; Reynolds et al., 2016) replacing the thymine in nuclear DNA, for transcriptional regulation and termination in kinetoplastid protozoans such as *Leishmania major, Leishmania donovani* and *Trypanosoma cruzi* facilitating intracellular parasite survival (Van Leeuwen et al., 1998). 5-hydroxymethyluracil is another epigenetic modification of thymine base alteration, which has been recently linked to the *Leishmania* genome. However, its epigenetic role in parasite survival has yet to be determined (Kawasaki et al., 2017).

Besides, a recent investigation pointed out a novel PTMs *via* bromodomain (BDs) complexes, mainly BD5. BD5 has emerged as a pivotal modulating factor in transcriptional factor of polymerase II in leishmanial parasite. Thus, BD5 can be used as an potential target to inhibit leishmanial infection by targeting the aforementioned mechanism to regulate transcriptional regulation in kinetoplastids (Jones et al., 2022). A plethora of investigation needs to be done in this direction to get an in-depth analysis of BDs based leishmanial regulation.

### 4.3 Epigenetics

The epigenetic markers such as H3K27ac, H3K9me3, H3K23ac H3K14ac promote transcriptional activation of ribosomal RNA (rRNA) genes in the promoter region of *L. major*, whereas H4K20me3 epigenetic marks in the coding region causes transcriptional silence (Vizuet-De-Rueda et al., 2016). H2A.Z and H2B.V histone variants are epigenetic markers found to be critical for *L. major* survival (Afrin et al., 2019). Histone acetyltransferase (HAT)4 acetylates H4K14 in *L. donovani*, promoting euchromatin maintenance (Yadav et al., 2016). *L. donovani* maintains its survival through epigenetic modulation via acetylation of H410 and H414 through HAT2 and HAT3 respectively (Chandra et al., 2017). To successfully establish itself inside the host, *L. donovani* utilizes epigenetic programming of genes in a histone lysine methyltransferase as well as demethylase dependent manner resulting in macrophage polarization in both J774 macrophages and BALB/c mice (Parmar et al., 2020).

In the promastigote and amastigote stages, epigenetic markers are regulated differently. Some histone deacetylases (HDAC) are preferentially increased in the logarithmic phase of promastigotes in *L. infantum* compared to intracellular amastigotes, allowing the amastigotes to adapt to the intra-phagolysosomal environment (Alcolea et al., 2010). Recent reports have indicated that NAD dependent HDACs sirtuins results in parasite persistence through inhibition of apoptosis. However, sirtinol causes apoptosis in axenic amastigotes of *L.infantum* (Vergnes et al., 2002; Vergnes et al., 2005).

A subset of non-coding RNAs is identified specifically in *L. infantum* and *L. donovani* amastigotes as a critical element for parasite survival within macrophages by causing modulation of epigenetics markers (Dumas et al., 2006). *L. donovani* causes epigenetic changes in host macrophages, irreversibly suppressing innate immune defenses (Marr et al., 2014). Upon *L. donovani* infection, several genes of signaling pathways such as JAK/STAT, calcium, MAPK, notch, mTOR and cell adhesion proteins (beta1 integrin) are differentially methylated at the CpG sites of the DNA resulting in increased oxidative phosphorylation (Lecoeur et al., 2022). Moreover, oxidative phosphorylation within the host increases the survival of the parasite by downregulation of innate immune response (Marr et al., 2014). The matrix metalloproteinase 1 (MMP1) gene which is involved in tissue repair gets downregulated by *L. braziliensis* causing lesions that facilitate the infection and ultimately lead to parasite survival (Wang et al., 2006; Almeida et al., 2015). Furthermore, epigenetic regulation by DNA methylation at the CpG promoters of the LOX gene upon activation by IL-6 in a homocysteine-dependent manner would lead to parasite survival (Thaler et al., 2011; Castellucci et al., 2014). The downregulation of iNOS by *L. amazonensis* in infected macrophages *via* histone deacetylase causes parasite survival (Calegari-Silva et al., 2018).

During *L. donovani* infection upregulation of miRNA-30A-3p causes parasite survival by targeting host autophagy machinery (Singh et al., 2016). The miRNA-3620 was found to influence iron homeostasis and hypoxia, while miRNA-3473f was associated with autophagy suppression in *L. donovani*-infected macrophages. The upregulation of miRNA-763, miRNA-1264, and miRNA-3473f causes drug resistance due to the overexpression of efflux pumps *via* ABC transporters (Singh et al., 2016; Tiwari et al., 2017). Thus, to establish a protective niche for its survival the leishmanial parasite causes a hypoxic environment within macrophages to regulate miRNA-210 in a HIF-1-dependent manner causing a pro-inflammatory response *via* regulating the NF-kB pathway (Kumar et al., 2018).

The leishmanial parasite influences the ncRNA (non-coding RNA) network *via* manipulation of the transcriptional arrest of the protein-coding genes of the macrophages for its survival (Rana et al., 2011). Downregulation of signal recognition particle RNA through ncRNA for parasitic survival inside the host is found in M2 macrophages by inhibition of RNA pol III (Misra et al., 2005). Additionally, *L. donovani* drives BRD4-p300-RNA-polII dependent unique super-enhancer element mediated miR146-5p transcription to promote parasite-supportive M2 macrophage niche (Das et al., 2021; Gupta et al., 2022). Gp63 and LPG are surface glycoproteins and glycolipids of *Leishmania*, shown to downregulate ncRNAs in M2 macrophages, thus helping in parasite persistence (Farrow et al., 2011). The overexpression of the host transcription factor c-myc leads to the downregulation of 19 miRNAs in infected macrophages with *L. donovani*. This would act as a prognostic marker for M2 macrophages for gene suppression causing parasite survival and proliferation. miRNA-34a which regulates the expression of c-myc is reciprocally downregulated in *Leishmania*-infected cells (Colineau et al., 2018).

### 4.4 RNA profiling and translational inducer

The level of proteins or translation rates can be assessed to study the effect of environmental stimuli on parasite gene expression (Bente et al., 2003; Rosenzweig et al., 2008a; Lahav et al., 2011), while the latter can be accomplished through ribosome profiling analysis or a combination of metabolic labeling/mass spectrometry method (Ingolia et al., 2009; Kalesh and Denny, 2019). The combination of ribosome profiling and RNA-Seq allows researchers to examine not only the relationship between mRNA abundance and translation but also the relative translational efficiency of mRNAs to environmental stimuli. Ribosome profiling paired with RNA-Seq revealed the inhibition of HSP90 to alter steady-state levels for mRNAs and cause increased or decreased protein synthesis rates for around 10% of the proteome in *L. donovani* (Bifeld et al., 2018). The early amastigote differentiation is enhanced when HSP90 is inhibited causing morphological alterations in the parasite (Lahav et al., 2011; Mandal et al., 2015; Bifeld et al., 2018).

Axenic amastigote differentiation is caused by decreased overall translation and phosphorylation of the translation factor eIF2a (Cloutier et al., 2012). Protein synthesis reduced in amastigotes causes reduced proliferation in the intracellular stage of the leishmanial life cycle (Jara et al., 2019). Under the influence of stress, there is a switch from cap-dependent to cap-independent translation in the leishmanial parasite to modify their translation machinery to environmental stress in a cap4, eIF2-alpha, eIF4E, and Leish4E-IP dependent manner (Zinoviev et al., 2011). Besides these discoveries, the involvement of translation factors in *Leishmania*’s inducible gene expression is unknown for their survival and drug resistance. Further studies in this direction needs to be done to get a proper understanding of the impact of translational inducer of leishmanial parasite survival.

## 5 Drug resistance in leishmanial parasite

### 5.1 Copy number variations (CNVs)

Even though CNVs are the most common form of resistance in the leishmanial parasite, SNPs are minor nucleotide insertions or deletions that cause drug resistance. *Leishmania* frequently uses DNA CNVs (aneuploidy, gene amplification, or gene deletion) to regulate the expression of drug targets, drug transporters, or drug resistance (Gordon et al., 2012; Pfau and Amon, 2012; Shor and Perlin, 2015; Rutledge and Cimini, 2016). Single-nucleotide polymorphisms (SNPs) caused due to CNVs in pharmacological targets or transporters lead to drug resistance without affecting gene expression (Alwi, 2005). SNP causes changes in the amino acid *via* substitution or non-sense mutations in the MTF transporter (MT) or its Ros3 subunit (Vasudevan et al., 2001; Pérez-Victoria et al., 2003; Pérez-Victoria et al., 2006; Laffitte et al., 2016c). This was further validated using whole-genome sequencing of MT (Coelho et al., 2012; Laffitte et al., 2016b; Mondelaers et al., 2016).

The drug resistance occurred primarily due to mutations in the MT gene of *L. infantum* isolates obtained from MTF-treated patients (Cojean et al., 2012). A genetic polymorphism in *L. donovani* isolates from the Indian subcontinent exhibited antimonial resistance *via* SNP (Imamura et al., 2016). Surprisingly, a group of highly resistant isolates clustering together were found to share genetic characteristics for drug resistance. The genetic characteristics for this drug resistance would include a high copy number of H-locus coding for ABC transporter MRPA and a homozygous two-base-pair insertion in the aquaglyceroporin 1 (AQP1) gene are involved for drug resistance in a Sb dependent manner (Légaré et al., 2001). Moreover, MRPA and AQP1 are involved in Sb uptake and their downregulation causes drug resistance both *in vivo* and *in vitro.* Thus, these Sb resistances may serve as a marker for the treatment of SNPs based on plasticity on a diverse set of isolates (Gourbal et al., 2004; Decuypere et al., 2005; Marquis et al., 2005; Uzcategui et al., 2008; Mandal et al., 2010; Mandal et al., 2015; Monte-Neto et al., 2015; Plourde et al., 2015).

### 5.2 Aneuploidy

The individual chromosomal ploidy is highly varied as it can occur between one to six sister chromosomes within leishmanial populations (Mannaert et al., 2012; Lachaud et al., 2014; Sterkers et al., 2014). Intermediate fold values of the NGS read alignment density can signal mosaic aneuploidy, such as the occurrence of population variations or incomplete chromosomal duplication occurrences (Imamura and Dujardin, 2019). Drug resistance *via* aneuploidy has been intensively investigated and due to decades of use of pentavalent antimony-based drugs in endemic areas, resistance has developed, making these drugs nearly obsolete in some locations (Dube et al., 2005; Croft et al., 2006; Singh et al., 2006; Ostyn et al., 2014). Advances are occurring in systems biology methodologies in *Leishmania* spp. for antimony resistance to aneuploidy patterns peculiar to resistant parasites (Leprohon et al., 2009; Laffitte et al., 2016c; Dumetz et al., 2017).

Mosaic aneuploidy plays a pivotal in vector and host adaptation owing to their diverse patterns of ploidy identified for vector and host-derived leishmanial cells grown *in vitro*. Ploidy changes occur *in vitro*, which produces clones with a ploidy pattern that differs from the parent (Sterkers et al., 2011; Sterkers et al., 2012; Dumetz et al., 2017). A recent study comparing ploidy and somy patterns of *L. donovani* strains was acquired through whole-genome sequencing (WGS) for parasite development in *in vitro* and karyotypic diversification. While cultured parasites possess a wide range of somy patterns, ploidies for bone-marrow-derived parasite genomes were strikingly identical (Domagalska et al., 2019), indicating similar selective pressures within human hosts. Despite this, *Leishmania* appears to be able to cope with genomic plasticity. Aneuploidy is easily reversed after the benefits of a supernumerary chromosome have worn off and when the overexpression owing to extra gene copies appears to be limited (Kröber-Boncardo et al., 2020). Regulated translation could thus help to alleviate the impacts of aneuploidy. Chromosomal amplification resulted in enhanced copy number of the gene which modulated the expression of the genes through host dependent environmental factor. The modulation in host dependent environmental factor prompted genetic tropism in species specific genes of leismanial parasites for genetic tropism as seen in species-specific genes 2, 14, 19, and 67 in *L. major, L. infantum*, and *L. braziliensis* (Rogers et al., 2011). This shows that aneuploidy is inherently unstable within a strain, fluctuating to changes in environmental factors causing plasticity for parasite survival (Rogers et al., 2011). Furthermore, extensive variation in the copy number of the chromosome for ploidy variability was concluded based on the WGS of varied strains of *L.donovani*. Thus, phylogenetically close strains (BPK173/Ocl3 and BPK275/Ocl18) exhibit a different somy for chromosomes 2, 8, 11, 12, 14, 20, and 33 owing to their heterogeneity of aneuploidy making it unstable over time. This ploidy instability explains the differences in the ploidy of the *L. major* Friedlin strain (2011) (Downing et al., 2011).

## 6 Future perspective and outlook

Even though drugs are introduced to the clinic without knowing more about their mode of action, a comprehensive understanding of the molecular targets for drug discovery is required. Target identification of possible resistance mechanism due to CNVs should be considered for drug use (Laffitte et al., 2016a; Paananen and Fortino, 2020). Genetic techniques experimentally mimicking CNVs for causing drug resistance and functional cloning were utilized for the gain-of-function screening method wherein leishmanial cosmid libraries were electroporated into *Leishmania sp* and transfectants for a specified phenotype (Ryan et al., 1993). The high copy number and gene expression of cosmids and selection of the phenotype were made possible for the selection of drug resistance or susceptibility. This method favors cosmids with dominant phenotypes while excluding less enriched cosmids (Vasudevan et al., 1998; Cotrim et al., 1999; Kündig et al., 1999; Carter et al., 2000; Coelho et al., 2003).

Amplification itself can produce drug resistance through different direct and indirect mechanisms. Interestingly, the artificial expression of cosmid-based functional screening coupled with a next-generation sequencing technique known as Cos-Seq has emerged as a novel technology that aids in the identification of target genes and their mechanism of drug resistance (Tejera Nevado et al., 2016) (Gazanion et al., 2016). Even though Cos-Seq identified an extraordinary number of resistance genes both known and novel but none appeared as an obvious target candidate. Thus, it is still unclear whether non-protein targets represent another possibility as these would not be detected by Cos-Seq and hence further research in this direction needs to be done.

The loss-of-function mutations in aquaglyceroporin AQP1 or MT transporter genes cannot be isolated using the Cos-Seq approach and require high-throughput dominant-negative screening methods through inducible RNA interference target sequencing (RIT-Seq) (Alsford et al., 2012). RNA interference is found to be absent in most of the leishmanial parasites but presents in *Leishmania (Viannia)* subgenus (Lye et al., 2010). Moreover, the lack of inducible expression in *Leishmania* (*Viannia)* was a drawback of this technique (Kraeva et al., 2014; Duncan et al., 2016). RNA-guided nuclease systems based on CRISPR has been developed as an alternative RIT-Seq for accomplishing effective specific genomic changes in a variety of organisms (Sander and Joung, 2014; Selle and Barrangou, 2015; Wright et al., 2016). The CRISPR/Cas9 system, derived from *Streptococcus pyogenes*, was used to disrupt genes in intracellular parasites such as *L. major* and *L. donovani*, and could be used to generate whole-genome Cas9-mediated gene deletion libraries (Sollelis et al., 2015; Zhang and Matlashewski, 2015). Thus, future research in this direction needs to be done to optimize this novel technology to be utilized against drug resistance to solve the issue of CNV in *Leishmania*.

## 7 Possible genomic variability mechanism

**Figure 2:**
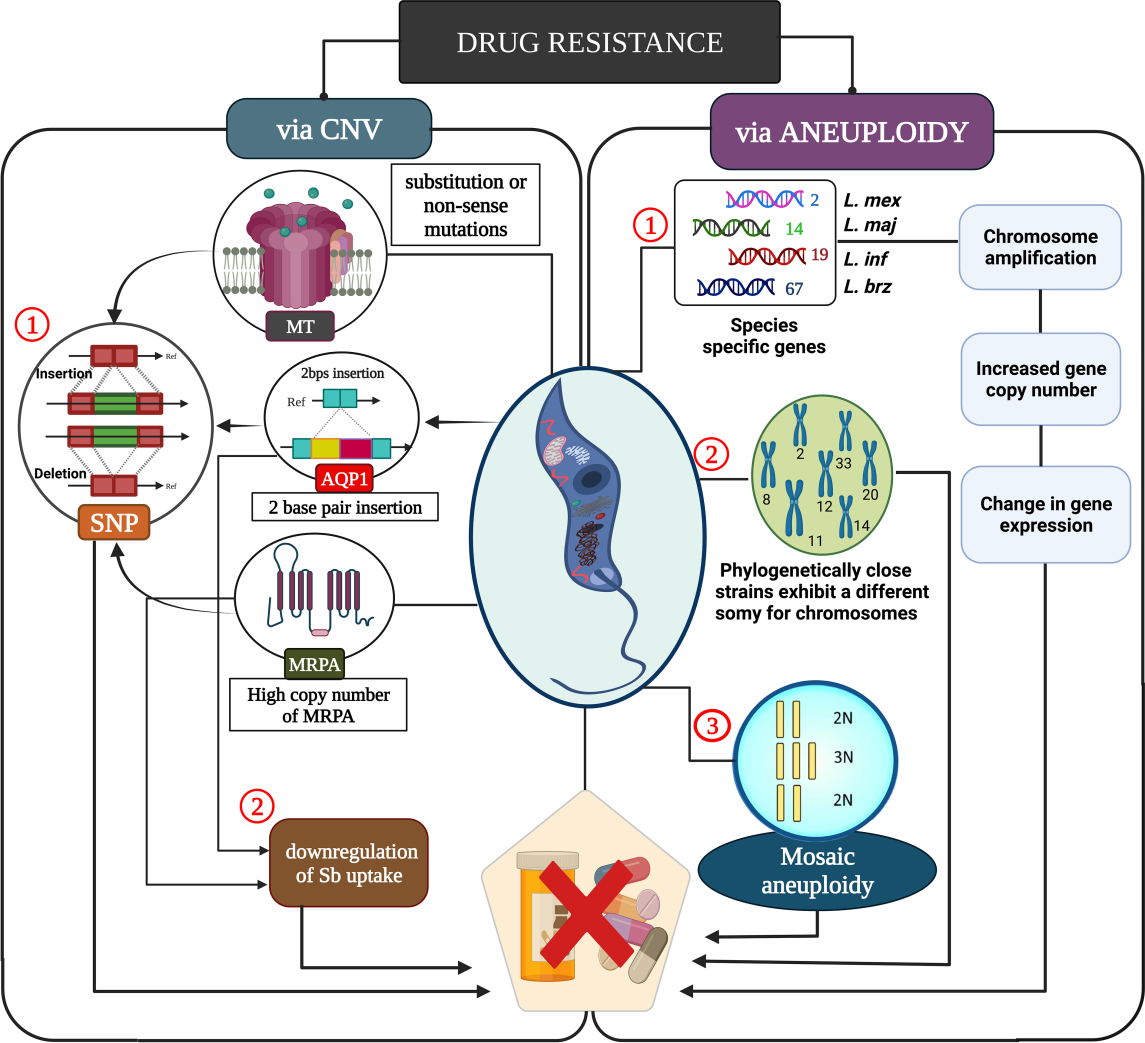
A molecular understanding of drug resistance caused by leishmanial parasite. Copy number variation influences drug resistance through single nucleotide polymorphism which affects the Sb uptake, ultimately leading to drug resistance. Mosaic aneuploidy causes increased copy number variation which leads to chromosomal amplification thus modulating the gene expression in several members of leishmanial parasites for drug resistance.

The understanding of genome plasticity at the molecular level in *Leishmania* opens a pathway to utilize such processes in combating drug resistance (Laffitte et al., 2016a) (**Figure 2**). Defective chromosomal replication through post-translational modification of replicative sequences as well as mis-segregation of the chromosome during mitosis results in mosaic aneuploidy as seen in these parasites (Zhu et al., 2018). Parasites amplify/delete gene copy number and such amplification of genes occurs *via* homologous recombination. DNA amplification occurs mainly when extrachromosomal elements are formed due to the rearrangement of direct or inverted homologous repeated sequences (Ubeda et al., 2008b). Genomic amplification of virulence factors such as GP63, Amastin, etc. increases the chance of survival of these parasites inside the host (Samarasinghe et al., 2018). *Leishmania* possesses unique replication machinery as there is a single polycistronic transcription unit with a single origin of replication in each chromosome which ultimately helps the parasite survive even under drug pressure (Downing et al., 2011). Leishmanial genes are constitutively expressed in these parasites by RNA polymerase II. In *Leishmania*, the promotor sequence is not yet deciphered properly, so the mechanism of transcription initiation is still under investigation (Grünebast and Clos, 2020b). Epigenetics mechanism seems to be involved by influencing DNA accessibility, which is an active area of research. It may be speculated that post-translational regulation of protein occurs through RNA degradation as there is a lack of proper transcriptional control. However, such studies show promise to decipher more about these mechanisms in the future.

## 8 Conclusion and summary

Successful treatment strategies for major public concerns like leishmaniasis are mainly jeopardized due to arising cases of resistant parasites. To survive in wide geographic real and different host species, *Leishmania* adapts its genome to be highly plastic. The genome plasticity is mainly maintained by aneuploidy, transposons, copy number variations, RNA trans-splicing of mono- and poly-cistronic mRNAs, and post-translational modifications. Hence, it is quite difficult to extrapolate and predict the role of essential proteins from general -omics data of *Leishmania*. The enormous plasticity of the genome not only helps parasites sabotage host machinery but also supports its survival under extreme drug pressure making new drug discovery quite difficult. Due to this, upon CNVs and PTMs prediction of promastigotes and amastigotes transcriptomics should be done cautiously as promastigotes may contain different sets of RNA expressions which can get modified and altered when they differentiate into amastigotes. Additionally, genome plasticity has a solid impact on drug metabolism and severely affects the outcome of advanced genome editing tools like RNAi, DiCre, plasmid shuffling, and CRISPR/Cas9 to understand their role in drug resistance. Our review has concisely described the set of proteins and epigenetic modifiers of parasites that contribute to CNVs and aneuploidy. Moreover, how such mediators can significantly bring about the unexpected outcomes of treatment cases with new anti-leishmanial agents and result in resistance generation can be an interesting area to explore. In a nutshell, our illustration depicts that an integrated understanding of the parasite proteins which mainly maintain the genome integrity and plasticity will assist in achieving successful chemotherapy against parasites like *Leishmania*.

## Author contributions

The manuscript was conceptualized by MK, RB, and NA. It was written and drafted by MK and RB. SD and SM helped in collecting and analyzing the data and proofreading the manuscript. NA guided the whole project and helped in funding acquisitions. All authors contributed to the article and approved the submitted version.
